# Proposal of the Implementation of an International Pharmacy Graduate Preliminary Examination

**DOI:** 10.3352/jeehp.2008.5.2

**Published:** 2008-12-22

**Authors:** Kyenghee Kwon, Jeoung Hill Park, Jinwoong Kim, Seung Ki Lee

**Affiliations:** College of Pharmacy, Seoul National University, Seoul, Korea.

**Keywords:** International Graduates, Pharmacy Schools, Pharmacist Licensing Examination, Preliminary Examination, Evaluation

## Abstract

At present, graduates of international pharmacy schools can apply to take the Korean Pharmacist Licensing Examination after passing a review by the Accreditation Board of the Pharmacy Schools and Licenses. However, since the educational content of different schools and the roles of pharmacists differ from country to country, a preliminary examination might be necessary before the Pharmacist Licensing Examination. To prepare to implement a preliminary examination for foreign pharmacy graduates in Korea, we summarized the preliminary examinations used in four other countries and presented a proposal for a preliminary examination. Data were collected via the internet and through telephone interviews with appropriate persons. The proposal was revised after a public forum. There are preliminary examinations in the USA, Canada, Australia, and the United Kingdom, and these involve written, oral, practice, and English proficiency tests. We proposed that the Korean preliminary examination consist of a written test on basic pharmacy, a test in the Korean language, and an interview. The preliminary examination should include suitable items that effectively evaluate international graduates. Graduates of international pharmacy schools who have an ability equivalent to graduates of Korean pharmacy schools should be eligible to write the Korean Licensing Examination.

## INTRODUCTION

In the era of the World Trade Organization (WTO) and free trade agreements (FTA), the mutual accreditation of medical health personnel is a topic of negotiation. Some nations have adopted preliminary or equivalency examinations for international graduates of pharmacy schools. For example, the USA limits candidates for the preliminary examination to graduates of pharmacy schools with 5-yr programs. Currently, graduates of international pharmacy schools can apply to sit the Korean Pharmacist Licensing Examination without taking part in an interview or preliminary examination, after passing a review by the Accreditation Board of the Pharmacy Schools and Licenses. However, since the educational content of international schools and the roles of pharmacists differ from country to country, a system of accreditation, such as a preliminary examination, might be necessary before allowing graduates of foreign schools to write the Pharmacist Licensing Examination. In Korea, pharmacists participate in the development, use, and evaluation of drugs, unlike some countries where pharmacists simply participate in the distribution and use of drugs. Although the Pharmacist Licensing Examination evaluates whether candidates have sufficient knowledge to work as pharmacists in Korea, other factors should be considered. In addition, the number of freshmen admitted to Korean pharmacy schools is limited to 1,300 by the Korean Government, as part of a national plan regulating the distribution of pharmacists. However, international graduates are not included in this national plan. Since approximately 60 international graduates now sit the Pharmacist Licensing Examination annually, it has not had a marked impact on the total number of pharmacists in Korea. However, if the number of international graduates increases, a preliminary examination should be used to control the quality of these graduates, as it might affect the manpower supply at the national level.

To prepare to implement a preliminary examination for graduates of foreign pharmacy schools who want to practice in Korea, we summarized the accreditation process in four other nations and presented a proposal for a preliminary examination.

## MATERIALS AND METHODS

The preliminary examinations used to evaluate pharmacists before they write the licensing examinations in the USA, Great Britain, Australia, and Canada were summarized [1-4]. Data were collected via the internet, by calling appropriate individuals, and through direct contact at international conferences. A proposal for the Korean preliminary examination was presented by the authors and revised based on opinions received at a public forum held on November 7, 2007.

## RESULTS

Preliminary examinations are held in the USA, Great Britain, Australia, and Canada, and include written, oral, practice, and English-proficiency tests. The various formats used in these countries are summarized in Table 1.

We suggest that the Korean preliminary examination consist of a test of proficiency in the Korean language, a written test on the basics of pharmacy, and an interview. The preliminary examination and the Pharmacist Licensing Examination are compared in Table 2 [5]. The test of proficiency in Korean may be replaced with the Test of Proficiency in Korean (5th grade) that is administered by the Korea Institute for Curriculum and Evaluation.

## DISCUSSION

Only a few OECD countries hold a preliminary examination before the respective Pharmacist Licensing Examinations in those countries. This suggests that the movement of pharmacists between most countries is infrequent. There may invisible obstacles to such movement, such as language barriers or a lack of economic benefit. There are no reports on a lack of pharmacists in specific countries. Therefore, unlike physicians or nurses, there is not believed to be an imbalance in supply and demand for pharmacists in most countries. In Korea, relatively few graduates of international pharmacy schools apply to write the Pharmacist Licensing Examination (less than 5% of all examinees); consequently, there is not much demand for a preliminary examination. In the future, however, more foreign graduates may apply to write the Korean Licensing Examination, so it is necessary to prepare a preliminary examination as a form of quality control.

We propose that the preliminary examination, if implemented, be launched in 2015, since it is the year when the first graduates of the 6-yr pharmacy course will write the Licensing Examination. At that time, it will be necessary to compare the abilities of graduates of Korean and international pharmacy schools. In order to implement this preliminary examination, the existing Pharmacy Law on eligibility for writing the Pharmacist Licensing Examination will also have to be revised. Suitable items for the preliminary examination should be constructed to enable an effective evaluation of international graduates. Equivalent ability between graduates of international and Korean pharmacy schools should be sufficient for eligibility to write the Licensing Examination. International graduates with acceptable results should be good candidates to write the Licensing Examination.

## Figures and Tables

**Table 1:**
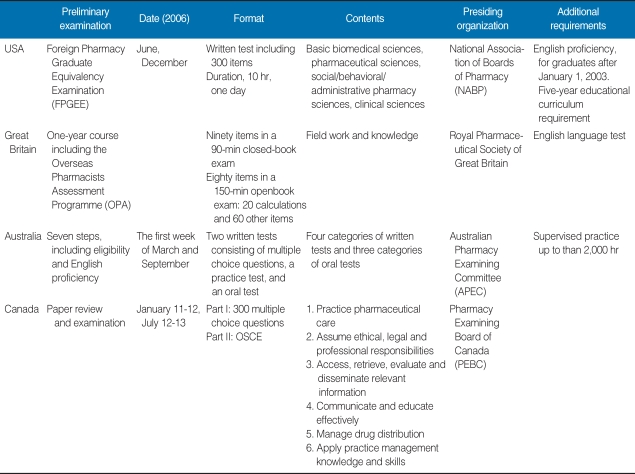
Preliminary pharmacy examinations held in four countries

**Table 2:**
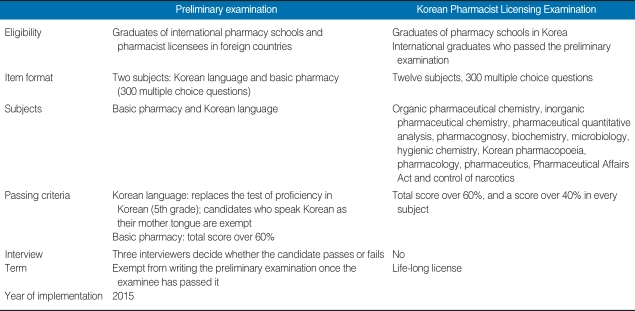
Comparison of the content of the preliminary examination and the Korean Pharmacist Licensing Examination
